# Satisfaction analysis of overground gait exoskeletons in people with neurological pathology. a systematic review

**DOI:** 10.1186/s12984-023-01161-4

**Published:** 2023-04-18

**Authors:** C. Cumplido-Trasmonte, F. Molina-Rueda, G. Puyuelo-Quintana, A. Plaza-Flores, M. Hernández-Melero, E. Barquín-Santos, MA. Destarac-Eguizabal, E. García-Armada

**Affiliations:** 1 Centre for Automation and Robotics (CAR), CSIC-UPM, Ctra Campo Real km 0.2 – La Poveda- Arganda del Rey, Madrid, 28500 Spain; 2 International Doctoral School, Rey Juan Carlos University, Madrid, 28922 Spain; 3 Department of Physical Therapy, Physical Medicine and Rehabilitation, Rey Juan Carlos University, Madrid, Spain; 4 Marsi Bionics S.L., Madrid, Spain; 5 Polytechnic University of Madrid, Madrid, Spain

**Keywords:** Overground exoskeleton, Satisfaction, Neurology, Rehabilitation

## Abstract

**Background:**

People diagnosed with neurological pathology may experience gait disorders that affect their quality of life. In recent years, research has been carried out on a variety of exoskeletons in this population. However, the satisfaction perceived by the users of these devices is not known. Therefore, the objective of the present study is to evaluate the satisfaction perceived by users with neurological pathology (patients and professionals) after the use of overground exoskeletons.

**Methods:**

A systematic search of five electronic databases was conducted. In order to be included in this review for further analysis, the studies had to meet the following criteria: [1] the study population was people diagnosed with neurological pathology; [2] the exoskeletons had to be overground and attachable to the lower limbs; and [3]: the studies were to include measures assessing either patient or therapist satisfaction with the exoskeletons.

**Results:**

Twenty-three articles were selected, of which nineteen were considered clinical trials. Participants diagnosed with stroke (n = 165), spinal cord injury (SCI) (n = 102) and multiple sclerosis (MS) (n = 68). Fourteen different overground exoskeleton models were analysed. Fourteen different methods of assessing patient satisfaction with the devices were found, and three ways to evaluate it in therapists.

**Conclusion:**

Users’ satisfaction with gait overground exoskeletons in stroke, SCI and MS seems to show positive results in safety, efficacy and comfort of the devices. However, the worst rated aspects and therefore those that should be optimized from the users’ point of view are ease of adjustment, size and weight, and ease of use.

**Supplementary Information:**

The online version contains supplementary material available at 10.1186/s12984-023-01161-4.

## Background

Stroke, spinal cord injury (SCI), multiple sclerosis (MS), Parkinson or cerebral palsy (CP) are some of the main causes of paresis around the world [1]. Lack of mobility and loss of independence to perform basic activities of daily living limit patients to a sedentary lifestyle, increasing the likelihood of chronic diseases [2]. Gait recovery in people with neurological disorders has a significant impact on quality of life and gait training is a relevant target of rehabilitation [3, 4].

In recent years, with the advent of improved electro-mechanical technology, faster data processing and the reduction of equipment size, exoskeletons have emerged as a new option in the field of rehabilitation that can enable walking around the environment [5]. Recent technology developments make overground gait exoskeletons increasingly available to rehabilitation facilities and patients with gait disorders. These technological innovations have become an alternative to manual gait rehabilitation [6]. Compared to conventional therapy, robotic gait rehabilitation can offer highly controlled, repetitive and intensive training in an engaging environment, reducing the physical burden on the therapist and providing objective and quantitative assessments of patient progression [7].

Currently, there are two types of exoskeletons for walking assistance in people with neurological pathology: body-weight-supported treadmill training (BWSTT) exoskeletons and overground exoskeletons [6, 8]. BWSTT exoskeletons included treadmill gait training, while overground exoskeletons included gait training that involved moving across the floor with or without body-weight support [9]. Recent narrative reviews [5, 10] of overground exoskeletons describe the current state of the art with device-specific features and limitations. Overground exoskeletons are emerging as revolutionary devices for gait rehabilitation due to the active participation required from the patient, which promotes physical activity [3, 11, 12], and the possibility of being used as an assistive device in the community [13]. Therefore, it is expected that the exoskeletons will be further developed to be used as a mobility device in the daily life by people with gait disorders [14].

Satisfaction can be defined as the extent to which the user’s physical, cognitive and emotional responses that result from the use of a system, product or service meet the user’s needs and expectations [15]. As research on wearable robotic exoskeletons in rehabilitation facilities has increased, recent studies have assessed patient and therapist satisfaction with the therapy. However, some studies refer that only 8% of the scientific literature regarding robotic exoskeletons has included considerations of patient satisfaction [16–18]. Considering that the satisfaction of an assistive device is highly dependent on the user’s perspective [19, 20], it is surprising that little research has focused on participant perceptions of these powered exoskeletons and their learning process [16–18]. Assessing overall patients and therapists satisfaction can help measure the aggregate quality of a product/service [21] and tracking patient satisfaction can help developers and researchers to improve the product/service for users. In patient engagement management, satisfaction is the extent to which a product/service meets patients’ expectations [22]. Focusing on the health care sector, quality of care and patient satisfaction are major issues [23]. Therefore, the evaluation of any device/service from patient’s and professional’s perspective is crucial [24–26].

In response to this lack of information about gait exoskeletons satisfaction, this paper aimed to systematically review the literature to assess the evidence concerning gait exoskeleton satisfaction in people with neurological pathology. The specific objectives are to: [1] Assess the satisfaction of gait overground exoskeleton interventions for people with neurological pathology and [2]: Describe the exoskeleton, participants and methodology used to assess satisfaction in this population. To the best of our knowledge, no such research has previously been carried out or published.

## Methods

### Selection process

The search focused on scientific articles which provided relevant clinical information aimed at studying participant or therapist’s satisfaction of interventions using a gait overground exoskeleton. In order to be included in the analysis, each article had to meet the following conditions: [1] the study population was people diagnosed with neurological pathology; [2] the exoskeletons had to be overground and attachable to the lower limbs; and [3]: the studies were to include measures assessing patient or therapist satisfaction or perception with the exoskeletons. There were no limitations regarding study design, year of publication, language, participants’ age or gender. Studies that used treadmills were excluded with the purpose of focusing only on studies that solely investigated overground exoskeleton effects without body weight support.

### Search strategy

We searched for scientific publications in five online databases: PubMed, Cochrane, Web of Science (WOS), Scopus and Springer Link using the following terms: (“usability” or “satisfaction”) AND “exoskeleton” AND “gait” AND (“stroke” or “spinal cord injury” or “multiple sclerosis” or “Parkinson” or “acquired brain injury” or “traumatic brain injury” or “cerebral palsy” or “neurology” or “neurological conditions” or “neurological pathology”). The term “usability” was used in the search strategy according to Earthy et al. [27], usability can be considered as “quality in use” and defined as “the extent to which a system, product or service can be used by specified users to achieve specific goals with effectiveness, efficiency and satisfaction in a specified context” (ISO 9241 − 210:2009). The final search was completed in February 2022.

The identification, screening and eligibility check of the studies were all done by the same author. In case of uncertainty during the screening or the classification process, a decision was reached in agreement with the authors of this manuscript.

### Methodological quality assessment

After determining which articles met the inclusion criteria, the same author conducted the level of evidence for the articles using the Oxford Centre for Evidence-Based Medicine (OCEBM) Levels of Evidence [28], which is characterized by assessing the evidence according to the subject area or clinical setting and the type of study involving the clinical problem in question. To assess methodological quality, a modified version of the quantitative McMasters Critical Appraisal Tool (MMCAT) [29] was used. The MMCAT assessed a range of domains as a part of the appraisal process including purpose, literature review, design, sampling, outcomes, intervention, results and conclusion with implications for practice. The original MMCAT was modified following the methodology used by Bunge et al. [30], in order to provide a numerical score as a result of the critical appraisal process. One point was given for every “yes” answer, while no points were obtained for “no” or “not addressed” answers. In some instances, the “N/A” criterion was selected (as the criterion did not apply to some study designs), which then altered the overall scoring. Therefore, the overall scores for individual studies were converted to percentages for easing the interpretation and comparison purposes.

The Preferred Reporting Items for Systematic Reviews and Meta-Analyses (PRISMA) guideline [31] was used as a quality control tool for the review.

### Data extraction and synthesis

The process of data extraction was performed by the same author. All the extracted data from studies were entered into tables for easy comparison and grouping. The following information was collected from the included studies: author and year details; exoskeleton used in the article; study design; sample characteristics; training details (number of sessions, frequency, and length); outcome measures used and results. If the data from any study was identified as missing, an attempt was made to contact the authors for the missing data, but if the authors did not respond, that information was not considered.

To ease understanding and facilitate easy comparison of satisfaction levels between different devices, a standardised score (%) is shown for the maximum score of each questionnaire. In the case that the results derived from the article only show averages score per item, the standardised score will be calculated using the maximum score of the items assessed.

## Results

**Fig. 1:**
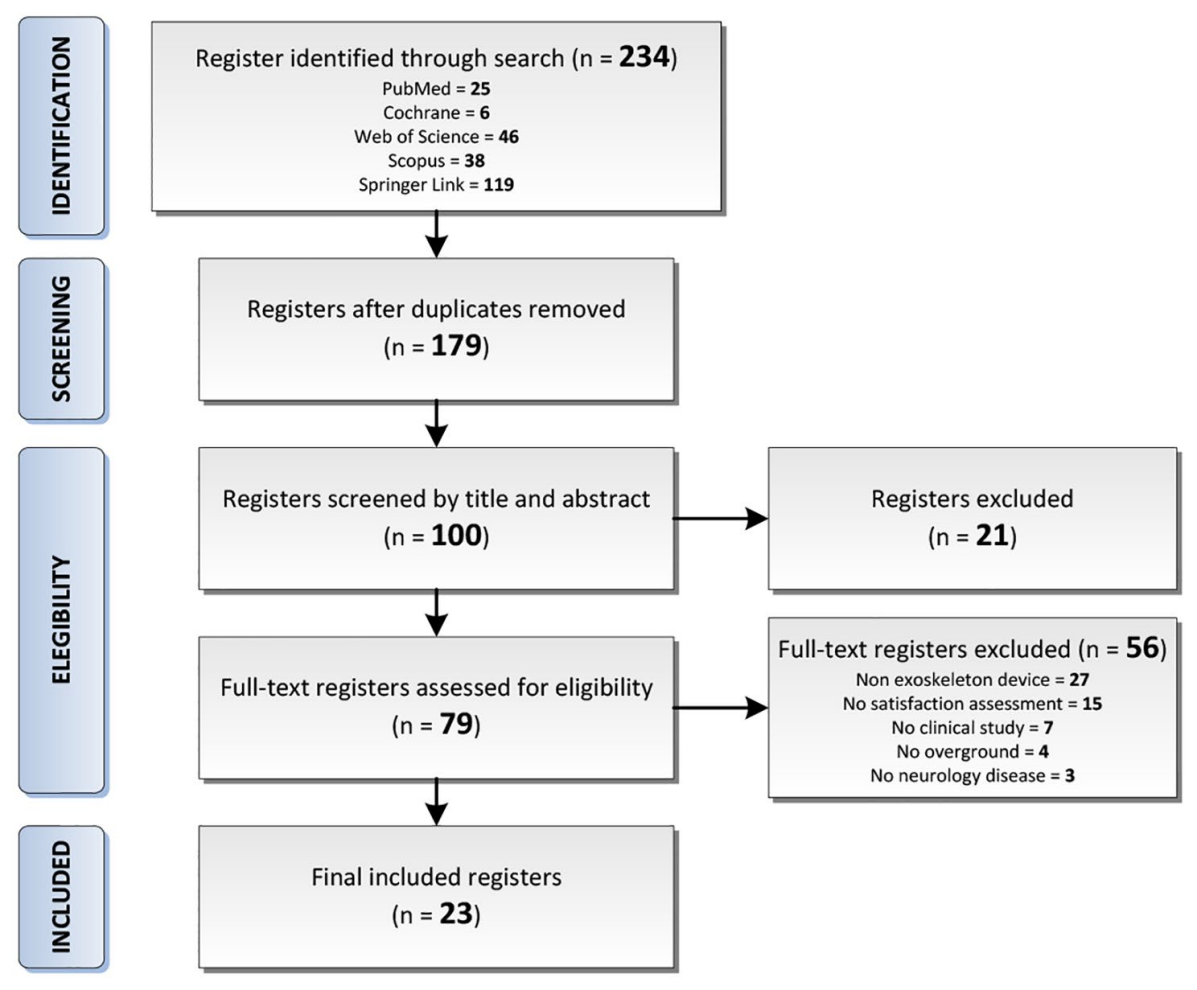
Flow diagram of the literature selection process according to PRISMA guidelines

**Fig. 2:**
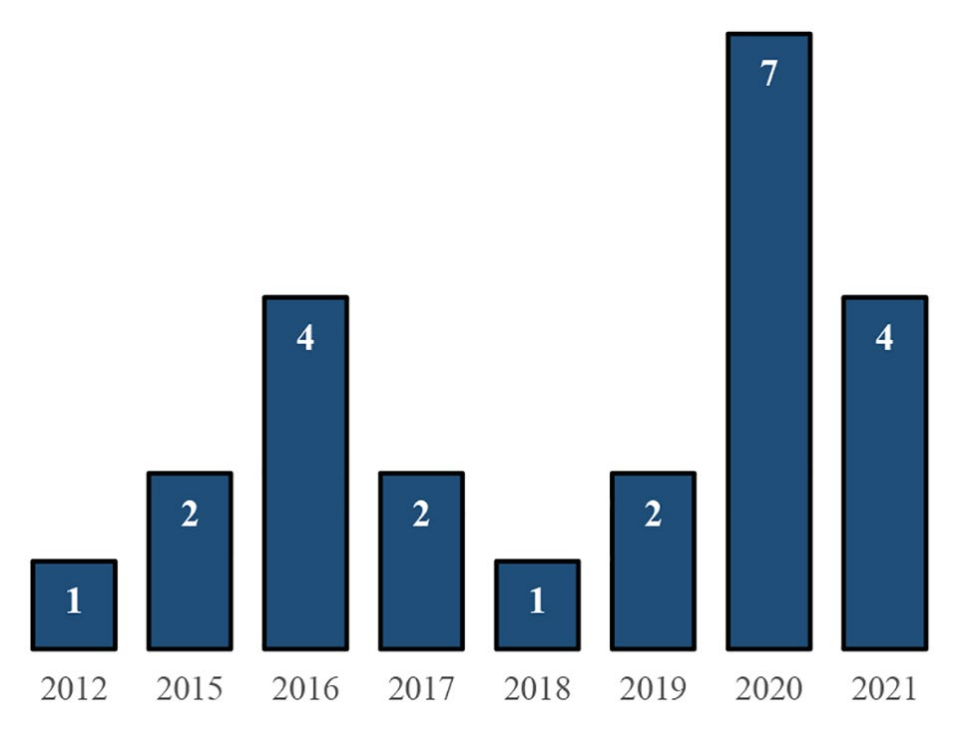
Studies included by publication year

The search strategy implemented generated a total of 234 articles in online databases. The bibliographic manager Mendeley® was used to eliminate duplicate articles, removing 55 studies from our search. Finally, the remaining 179 publications were screened by their title and abstract according to the PICO model. 100 publications were full-text assessed for eligibility and 23 were finally selected (Fig. 1) to be analysed in detail (Table 1). Selected studies were published between 2012 and 2021 and all included studies were written in English. In total, there were 11 studies [13, 32–41] that included participants with SCI (n = 95), 8 studies [42–49] included participants diagnosed with stroke (n = 146), two studies [50, 51] included participants with MS (n = 67), 1 study [52] participants with SCI and stroke (SCI n = 7 and stroke n = 16), and other [53] with stroke and MS (stroke = 3, and MS = 1). In addition, in Fig. 2 is showed the volume of analysed studies per year.

**Table 1:** Summary table of included studies

Author and year	Exoskeleton	Pathology	N	Sessions	Satisfaction measure	Main satisfaction results	Normalized score (%)
Awad 2020 [46]	ReStore	Stroke	44	8	QUEST 2.0 Physical Therapist Questionnaire	Overall participant satisfaction: 33.8 ± 6.1 out of 40. Parameters consider by participants more relevant: effectiveness (69.4%), comfort (52.8%) and ease of use (50.0%). Overall physiotherapist’s satisfaction: 33.7 (maximum score: 45). Less valued parameters by therapists: donning/doffing time (3.1 ± 0.95), usefulness in clinical practice (3.4 ± 0.9) and recommendation to other professionals (3.4 ± 0.9). Most valued by physiotherapists: ease of use (4.3 ± 0.9) and user interface (4.3 ± 0.8).	P 84.5 T 74.9
Birch 2017 [39]	REX	SCI	20	1	Device Acceptability Questionnaire (REX)	95% of participants felt very confident and stable, 89% very safe. 78% strongly agreed that they enjoyed their experience and would use it weekly and would recommend. Sound and size of REX influenced 8 participants negatively. 98% of participants would like the device to be more affordable.	P 83.4
Bortole 2015 [48]	H2	Stroke	4	12	Likert ease of use	Overall rating: 7.2 out of 10, were 0 indicates “extremely hard to use” and 10 indicates “extremely ease of use”.	P 72.0
Chihara 2016 [47]	HAL	Stroke	15	6	Satisfaction interviews family and patients	No satisfaction results.	-
Corbianco 2021 [38]	Ekso GT	SCI	15	17	Subjective Experience Participant Impact	Ekso overall satisfaction and emotion: 6.6 ± 2.2 and 5.0 ± 3.3 out of 10. Low scores of fatigue, mental effort and fear or discomfort were observed in the Lokomat. Lokomat was perceived to be less demanding when compared to the Ekso training.	P 52.7
Del-Ama 2015 [41]	Kinesis	SCI	3	3	QUEST 2.0	The overall satisfaction was 32.0 (4.0 ± 0.9) out of 40. The overall lowest scores: weight (3.3 ± 0.4), fitting (3.2 ± 0.5) and comfort (3.0 ± 0.0) whereas safety (4.25 ± 0.9), durability (4.5 ± 0.5) and efficacy (5.0 ± 0.0) were top rated.	P 80.0
Dijsseldonk 2020 [13]	ReWalk	SCI	14	1 to 15	QUEST SUS	Overall participant satisfaction: 3.7 ± 0.4 per item out of 5. Subscale assistive device 3.5 ± 0.4 and 4.2 ± 0.5 in subscale service. Weight, Effectiveness, Ease of use and Safety were the most frequently scored as dissatisfied (mean < 3.0), and at the same time indicated as important. The mean SUS score was rate with a median of 72.5% [52.5–95.0]. Low usability (< 3.0) in: ease of use and device function integration.	P 74.0*
Esquenazi 2012 [37]	ReWalk	SCI	12	24	Participant Satisfaction Questionnaire	27.7% of participants reported improved spasticity. No participant felt any pain. 9% of subjects fatigue. 45.5% of subjects reported improved bowel regulation.	-
Fernández-Vázquez 2021 [50]	Ekso GT	MS	40	13	QUEST 2.0 Client Satisfaction Questionnaire (CSQ-8) Physical Therapist Questionnaire	Overall QUEST satisfaction: 31.3 ± 5.7 out of 40. Parameters considered more relevant: effectiveness (32%), safety (26%) and ease of use (22%). Overall CSQ-8 satisfaction: 26.3 ± 4.7 out of 32 points. Correlation between number of sessions and patients’ satisfaction (rho = 0.5; p < 0.001). Overall physiotherapist’s satisfaction: 38.5 ± 3.7 out of 45 points. Correlations: age and satisfaction device adjustment (rho = 0.7; p = 0.007); and between experience and satisfaction combining with other gait trainings (rho = 0.7; p = 0.003).	P 78.2 T 85.5
Gómez-Vargas 2021 [45]	T-FLEX	Stroke	10	1	QUEST 2.0	Parameters more relevant: comfortable (70%); safety (60%); weight (60%). Satisfaction level between satisfied (60%) and very satisfied (40%).	P 33.3
Høyer 2020 [44]	Ekso GT	Stroke	26	9	Likert satisfaction, usefulness, disadvantages, and willingness to repeat.	Overall satisfaction and usefulness of the training sessions 5.0 out of 5. 1.0 out of 4 no inconveniences as a result of the training and willingness to repeat exercises with Ekso GT.	P 100*
Jyräkoski 2021 [49]	Indego	Stroke	5	16	Participant Satisfaction Questionnaire + 2 additional questions	Overall satisfaction: 35.6 out of 50 (3.5 ± 0.6). The lowest scores: improvements in bowel movement (2.4 ± 1.1), and ease of adjusting the device (3.8 ± 0.7). The best rated: comfort during and after the session (4.2 ± 0.8 and 4.2 ± 0.5). Willing to use it as rehabilitation tool in the future in 80% of participants, and none of them could imagine using it at home.	P 71.2
Kozlowski 2017 [51]	ReWalk	MS	8	20	QUEST 2.0	The average total of the QUEST 2.0 was 29.3 ± 2.5 (range 2.8 ± 0.4 to 5.0 ± 0.0).	P 73.2
Kwon 2020 [36]	ReWalk	SCI	10	20	Usability evaluation questionnaire of walking devices (auto developed) Interview	The usability of a ReWalk + crutches was compared with a KAFO + Walker. KAFO best rated compared to ReWalk on: safety (3.5 ± 0.6 vs. 3.3 ± 0.8); effectiveness (3.5 ± 0.7 vs. 3.2 ± 0.6); efficiency (3.3 ± 0.7 vs. 3.0 ± 0.5); and overall satisfaction (4.2 ± 0.8 vs. 3.5 ± 0.7).	P 65.2*
López-Larraz 2016 [32]	H2	SCI	4	3	QUEST 2.0	Overall satisfaction: 30.5 out of 45 (3.3 ± 0.6). The lowest scores: comfortability (2.5), weight and ease in adjusting (3.0). The highest scores: safety and ease of use (4.2), and effectiveness (3.7).	P 67.8
Nam 2019 [43]	Exowalk	Stroke	18	20	Questionnaire on satisfaction of electromechanical exoskeleton-assisted gait training	Overall satisfaction was 4.1 ± 0.2 out of 5.0. High satisfaction rates: improvement of depression (4.5 ± 0.5), confidence in gait (4.2 ± 0.9), and desire to continue gait training (4.7 ± 0.6). The lowest score: motivation (3.8 ± 0.9).	P 82.0*
Platz 2016 [35]	ReWalk	SCI	7	25	Participant Satisfaction Questionnaire	The lowest score: spasticity (2.1), followed by fatigue (2.5) and ease of use (2.9). The best rated: breathing difficulty (4.9), pain (4.8) and comfort after the end of the session (4.6) out of 5.0.	P 42.0
Puyuelo-Quintana 2020 [53]	MAK	Stroke MS	3 1	1	QUEST 2.0	Overall satisfaction: 22.4 ± 3.2 out of 40 (2.8 ± 0.4). The best features of the exoskeleton: safety, size and weight (3.6). The lowest scores: effectiveness (2.4); and ease of use (2.6) and durability (2.6). The lowest score was the effectiveness of the device in resolving the participant’s problems.	P 56.0
Sale 2016 [33]	Ekso	SCI	3	20	Participant Satisfaction Questionnaire	Two satisfaction assessments: at the start of the study (T0) and after 20 sessions of use (T1). Overall total at T0: 43.6 (4.3 ± 0.6) and at T1: 45 out of 50 (4.5 ± 0.3). The biggest improvements compared to T0: improvement bowel movement and safety (up 0.7 and 1.6 points respectively). The best T1 scores: improvement on spasticity (5.0 ± 0.0) and breathing difficulties (5.0 ± 0.0). The lowest score: fatigue (4.0 ± 1.0).	T0 87.2 T1 90.0
Sale 2018 [34]	Ekso	SCI	8	20	Participant Satisfaction Questionnaire	Two satisfaction assessments: at the start of the study (T0) and after 20 sessions of use (T1). The overall total at T0 was 39.9 out of 50 (3.9 ± 0.6) and T1 was 45.1 out of 50 (4.5 ± 0.4) points. After T1 all items increased in score. All scores greater than 4.0, with the exception of fatigue (3.7 ± 1.2), and 5 points were scored on improvement of spasticity and respiratory distress.	T0 79.8 T1 90.2
Swank 2020 [52]	Ekso GT	Stroke SCI	16 7	8 4	Therapist Interview Ekso therapist feasibility Ekso patient feasibility	Feedback from therapists improved after six months except for communication between therapists and the Ekso trainer. All dimensions exceeded 70% scale score (2.9), with best results in overall satisfaction with therapy (3.7). No results were shown by pathology.	P 83.1*
Tamburella 2020 [40]	Achilles	SCI	4	10	QUEST 2.0	2 participants very satisfied (5.0) for all the aspects, with the exception about Achilles donning/doffing procedures (2.0) and reliability/robustness (2.0). Other 2 participants satisfied (4.0) by dimension, safety and ease of use.	-
Villa-Parra 2019 [42]	ALLOR	Stroke	3	1	QUEST 2.0	Overall satisfaction per item 4.2 ± 0.4. All categories exceeded 3.9 score, with a score of 5.0 for ease of use. The best feature of the exoskeleton was ease of use (5.0 ± 0.0 and the lowest scores were: dimensions (3.9 ± 0.0); weight (3.9 ± 0.8); and effectiveness (3.9 ± 0.8).	P 84.0*

N: Number of participants; SCI: Spinal Cord Injury; QUEST: Quebec User Evaluation of Satisfaction with Assistive Technology; SUS: System Usability Scale; MS: Multiple Sclerosis; CSQ-8: Client Satisfaction Questionnaire; KAFO: Knee Ankle Foot Orthosis; MAK: Marsi Active Knee; P: patient; T: therapist. * Denote calculations based on the maximum score per item.

### Synthesis of the included studies

The sample size of the included articles was 8.9 ± 5.4 (range 3–20) people for SCI studies, 14.5 ± 12.8 (range 3–44) people for stroke studies and 16.3 ± 20.8 (range 1–40) people for MS studies. The included studies enrolled a total of 107 participants with SCI, 145 with stroke and 49 with MS. Male participants with SCI and stroke were 68.2% and 63.4% respectively, while in MS studies the sample was split 50% according to the gender. The age range of SCI participants was 16–68, 17–83 for stroke and 38–62 years for MS. The range of time since injury varied from 7 days to 29 years in people with SCI and from 3.6 days to 32 years in people with stroke.

Regarding the levels of SCI, the participants were: 11% cervical level; 25% thoracic level (Th) 1–6; 46% Th7-12 level and 18% lumbar level. And the classification of the participants according to the AIS scale was: 52% A; 18% B; 18% C; and 5% D. Regarding the classification and/or functionality of participants with stroke or MS, no homogeneous classification methods or descriptions were found that considered the origin or functionality of the stroke, or the type or functionality of MS.

This review identified 14 exoskeletons from which nine have FDA approval and/or CE certificate and are commercially available: ReWalk™ (ReWalk Robotics, Inc. USA); ReStore™ (ReWalk Robotics, Inc. USA); Ekso™ (Ekso Bionics Inc, USA); Ekso GT™ (Ekso Bionics Inc, USA); Indego™ (Parker Hannifin Corporation, Human & Control, USA); HAL™ (Cyberdine, Inc, Japan); REX™ (REX Bionics Ltd, New Zealand); MAK™ (Marsi Bionics S.L., Spain); and Exowalk™ (HMH Corp, Korea). The other exoskeletons included in the studies were: T-FLEX, ALLOR, Achilles, Kinesis and H2. The following exoskeletons were evaluated in more than one study: ReWalk (n = 5), Ekso GT (n = 5), Ekso (n = 2) and H2 (n = 2).

We found that 9 of 14 exoskeletons (64%) were completely bilateral and 8 exoskeletons actively assisted two or more joints (5: hip-knee, 3: hip-knee-ankle), while the rest actively aids in a single joint (3: knee, 3: ankle). The number of actuated degrees of freedom (DOF) in included exoskeletons ranges from one to three per leg in the sagittal plane (except for REX, which also enables movement in transverse and coronal planes).

The control strategies used in the exoskeletons included were mostly through assist-as-needed with stiffness variations algorithms, defined as the device ability to include the user’s capabilities as part of its control strategy. The exoskeletons that do not use this control system are Rewalk™ and REX, the latter having obstacle avoidance capabilities.

**Fig. 3:**
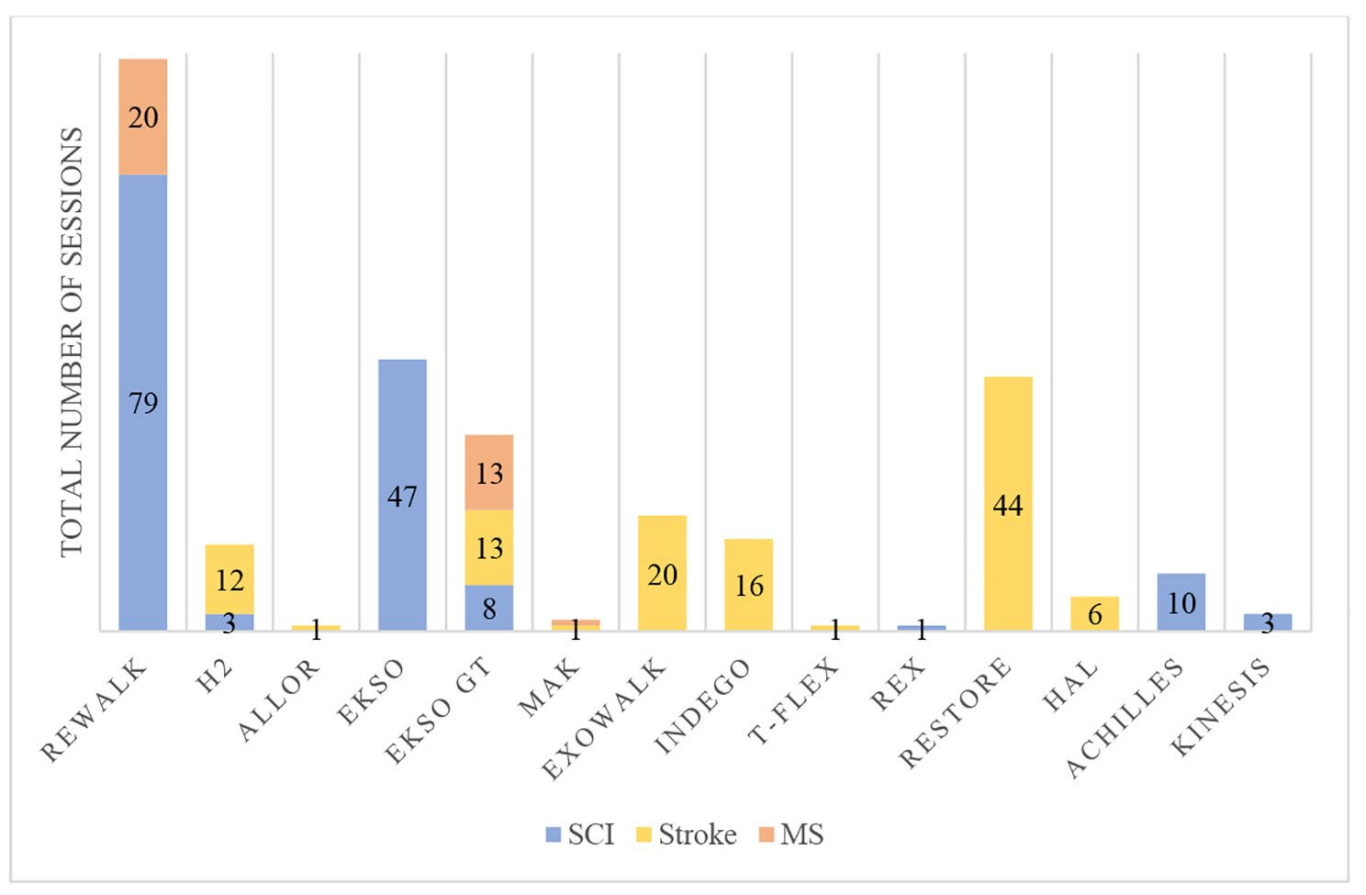
Total number of sessions distributed by exoskeleton and pathology

Every study analysed was carried out in hospital facilities, except for one intervention that was carried out in the community (outdoor and indoor) and at home on a free-to-use basis [13]. The total number of sessions performed with the exoskeletons ranged from one [39, 42, 45, 53] to 25 for SCI [35] and 20 for stroke and MS [43, 51]. Total study duration ranged from 1 day [39, 42, 45, 53] to 62 days for SCI [52] and 56 for stroke [49], with a weekly frequency between 0.8 [52] and 0.9 [52] sessions for stroke and SCI respectively and 5 sessions per week [35, 36, 43]. The time of use of the devices in the sessions of the selected studies ranged from 6 min [41] to 75 min [36] for SCI and between 27.5 [52] and 60 min per session for stroke [44, 49]. See Fig. 3 for the total number of sessions performed per exoskeleton and pathology.

Regarding to the tools used to assess the participant and therapist satisfaction with the exoskeletons, the included articles used 14 different methods. The most commonly used questionnaires to assess participant satisfaction were the Quebec User Evaluation of Satisfaction with Assistive Technology (QUEST 2.0.) [13, 32, 40–42, 45, 46, 50, 51] and the Participant Satisfaction Questionnaire (PSQ) [33–35, 37, 49] used in 44% (n = 10) and 22% (n = 5) of the included studies, respectively. One of the included studies used the QUEST questionnaire with the services Sect. [13]. One of them added two additional items to the PSQ: “*I could imagine using the device as an assistive walking aid at home*” and “*I would use the device as rehabilitation device in the future*” [49].

Two studies [39, 52] used questionnaires developed by the company that produces the exoskeletons (Ekso, REX), one study developed a specific questionnaire for their study [36], other study used the Client Satisfaction Questionnaire (CSQ-8) [50], two conducted interviews with relatives [47] and patients [36, 47], and several assessed satisfaction parameters using Likert scales of: satisfaction, usefulness, disadvantages, willingness to repeat [44] and ease of use [48].In general, the issues most frequently evaluated through the participant satisfaction questionnaires were parameters related to: effectiveness (22% of the items in all questionnaires), overall satisfaction and recommendation (17%), ease of use (12%) and safety (10%).

Only three of the included studies assessed therapists’ satisfaction with the device [46, 50, 52]. The studies used different tools: an interview [52], the Physical Therapist Satisfaction Questionnaire (PTSQ) [46, 50] and the Ekso therapist feasibility survey [52], which was more focused on assessing the knowledge acquired after training and ease to use of the device. The PTSQ assessed aspects such as the amount of time to adjust the device and settings, safety, understandable device feedback and therapist satisfaction with the device.

Overall, the most frequently evaluated aspects of the therapists’ questionnaires were related to: ease of use (33% of the items in all questionnaires), ease of adjustment (17%), and effectiveness (17%). Other aspects assessed in the questionnaires were fatigue, safety and overall satisfaction with the device.

### Main results: participants’ satisfaction with overground exoskeletons

One study did not provide its results on satisfaction, but the authors decided to include it in the analysis of the review in order to specify its satisfaction assessment methodology [47]. Participants’ satisfaction with the use of the exoskeleton in people with SCI, stroke and MS was generally positive, exceeding the average cut-off score in all questionnaires. The worst evaluated characteristics of the devices, and therefore the aspects to be optimised for development were: ease of adjustment and removal of the device [36, 40, 41, 46, 49], size and weight [13, 36, 39, 41, 42], and ease of use [13, 35]. On the other hand, the most positive exoskeletons features were: safety [32, 41, 46, 53], efficacy [41, 44] and comfort [35, 49, 53].

Concerning the QUEST questionnaire, the average score per item was 3.7 ± 0.5 points out of 5.0. The average per pathology was: stroke 3.7 ± 0.8 [42, 46, 53]; SCI 3.7 ± 0.3 [13, 32, 41]; and MS 3.7 ± 0.2 [50, 51]. The three parameters considered most important in a overground exoskeleton in the QUEST questionnaire were: effectiveness [13, 46], ease of use [13, 46], safety [13, 45], weight [13, 45] and comfort [45, 46].

Regarding the participants satisfaction questionnaire, which was shown just by the average results per item for the SCI studies, 4.1 ± 0.6 points per item [33, 34, 49] out of 5.0 were obtained. Participants self-reported improvements in spasticity [33, 34, 37], in bowel regulation [33, 37, 49] and in breathing difficulties [33–35]. These last three issues were included in the analysis of this review because they were part of the participants’ self-perceived satisfaction questionnaires, and not as outcomes related to the efficacy assessment by professionals.

Only two studies showed quantitative data regarding therapist satisfaction using the physical therapist questionnaire, in stroke the average was 3.7 out of 5.0 per item [46], and in MS was 4.3 out of 5.0 per item [50]. Difficulties related to donning/doffing were shown in the three studies that evaluated therapist satisfaction [46, 50, 52].

### Methodological quality of the included studies

There were 19 studies identified as clinical trials according to the Clinical Trial definition proposed by the National Institutes of Health (NIH) [54] (see Additional file 1 for a detailed view on the clinical trial identification assessment). Table 2 provide an overview of the methodological quality and the level of evidence of the included studies. Most of the studies in this review are Non-Randomized Studied of Interventions (NRSI).

**Table 2:** Levels of evidence and critical appraisal scores

Study	OCEBM level and study design	Items on modified McMaster critical appraisal tool (MMCAT)														
		**1**	**2**	**3**	**4**	**5**	**6**	**7**	**8**	**9**	**10**	**11**	**12**	**13**	**14**	**Raw score and %**
Awad 2020 [46]	IV; Case series	1	1	1	0	1	1	1	N/A	0	1	1	1	1	0	10/13; 76.9%
Birch 2017 [39]	IV; Cohort	1	1	1	0	0	1	1	N/A	N/A	0	1	1	N/A	1	8/11; 72,7%
Bortole 2015 [48]	IV; Case series	1	1	1	0	0	1	1	N/A	0	0	1	0	0	1	7/13; 53.8%
Chihara 2016 [47]	IV; Case series	0	0	1	0	0	1	0	N/A	0	0	0	0	1	0	3/13; 23.1%
Corbianco 2021 [38]	IIB; RCT	0	1	0	0	0	1	1	0	0	1	1	1	0	1	7/14; 50.0%
Del-Ama 2015 [41]	IV; Case series	1	1	1	0	1	1	1	N/A	0	0	1	0	1	1	9/13; 69.2%
Dijsseldonk 2020 [13]	IV; Case series	1	1	1	0	1	1	1	N/A	0	0	1	1	N/A	1	9/12; 75%
Esquenazi 2012 [37]	IV; Case series	1	1	1	0	1	1	1	N/A	0	0	1	1	0	1	9/13; 69.2%
Fernández-Vázquez 2021 [50]	IV; Cross-sectional	1	1	1	1	1	1	1	N/A	0	1	1	1	N/A	1	11/12; 91.7%
Gómez-Vargas 2021 [45]	IV; Case series	1	1	1	0	1	1	1	N/A	N/A	1	1	1	N/A	1	10/11; 90.9%
Høyer 2020 [44]	IV; Case series	1	1	1	0	0	1	0	N/A	0	1	1	1	1	1	9/13; 69.2%
Jyräkoski 2021 [49]	IV; Case series	1	1	1	0	1	1	1	N/A	0	0	1	1	1	1	10/13; 76.9%
Kozlowski 2017 [51]	IV; Case series	1	1	1	0	1	1	1	N/A	0	0	1	1	1	1	10/13; 76.9%
Kwon 2020 [36]	IV; Cross-sectional	1	1	1	1	1	1	1	N/A	0	0	1	1	0	1	10/13; 76.9%
López-Larraz 2016 [32]	IV; Case series	0	1	1	0	1	1	1	N/A	0	0	1	1	0	0	7/13; 53.8%
Nam 2019 [43]	IB; RCT	1	1	1	1	1	1	1	0	0	1	1	1	0	0	10/14; 71.4%
Platz 2016 [35]	IV; Case series	1	1	1	0	1	1	1	N/A	0	0	1	1	1	1	10/13; 76.9%
Puyuelo-Quintana 2020 [53]	IV; Cross-sectional	1	1	1	0	1	1	1	N/A	N/A	0	1	1	N/A	1	9/11; 81.8%
Sale 2016 [33]	IIIB; Case series	0	1	1	0	1	1	1	N/A	0	1	0	1	1	0	8/13; 61.5%
Sale 2018 [34]	IV; Case series	1	1	1	0	1	1	1	N/A	0	1	1	1	1	0	10/13; 76.9%
Swank 2020 [52]	IIB; Cohort	1	1	1	0	0	1	0	N/A	0	1	1	1	0	0	7/13; 53.8%
Tamburella 2020 [40]	IV; Case series	1	1	1	0	1	1	1	N/A	0	0	1	1	1	1	10/13; 76.9%
Villa-Parra 2019 [42]	IV; Case series	0	0	0	0	1	1	0	N/A	N/A	0	1	0	N/A	0	3/11; 27.3%

MMCAT items to be scored: (1) Was the purpose stated clearly?; (2) Was relevant background literature reviewed?; (3) Was the sample described in detail?; (4) Was the sample size justified?; (5) Were the outcome measures reliable?; (6) Were the outcome measures valid?; (7) Intervention was described in detail?; (8) Contamination was avoided?; (9) Cointervention was avoided?; (10) Results were reported in terms of statistical significance?; 11. Were the analysis method/s appropriate?; 12. Clinical importance was reported?; 13. Drop-outs were reported?; 14. Conclusions were appropriate given study methods and results?. 1 = yes, 0 = no or not addressed, N/A = not applicable. OCEBM: Oxford Centre for Evidence-Based Medicine Levels of Evidence.

Only three Randomized Controlled Trials (RCT) were found [36, 38, 43]. 86% of the included studies had IV level (out of five) of methodological quality according to the OCEBM [28]. The methodological quality of the included studies was considered as poor to moderate. The raw scores were converted to a percentage for ease of comparison across the included studies as some criteria did not apply to all study designs. For example, the criterion for contamination was no applicable because the majority of the included studies were either cases series or case studies, there were no control or comparator groups. The highest critical appraisal was awarded to Fernández-Vázquez et al. [50] and the lowest to Chihara et al. [47]. While most of the included studies scored well for criterion two, three, six, eleven and twelve, there were a number of methodological concerns. These included measurement bias due to lack of sample size justification (criterion 4) and co-intervention bias (criterion 9). While the lack of participants’ blinding, therapists and measures increases the risk of placebo, Hawthorne effect [55] and measurement bias, given the nature of the intervention, these biases could not be entirely avoidable.

## Discussion

The main objective of the present study is to assess satisfaction with gait exoskeletons in people with neurological pathology. This objective is particularly relevant in the field of exoskeleton development since feedback from patients and therapists is essential for further device optimization. In addition, the positive and negative aspects are critical to consider from the design stage and can be part of the technical requirements. This impacts not only companies that already have exoskeletons on the market, but also start-ups in the product development phase and research groups working in this field. However, regarding the articles published on gait exoskeletons, only few papers show the analysis of this key variable [16–18]. This lack of information in this field may also be due to confidentiality within these companies, whose aim could be to improve the device compared to the competitors, making this information highly sensitive to potential competitors in this technological field. Nevertheless, it is important to note that there seems to be an upward trend in the number of studies analysing satisfaction variables, with 70% of the studies included in this study having been published in the last 5 years. This increase of satisfaction-related studies may also be due to the exponential growth of publications concerning gait exoskeletons in recent years [12].

The search of published papers was carried out to find a variety of neurological pathologies, however only three were found: stroke, SCI and MS. Although it is true that these pathologies are the most studied in gait exoskeletons, there is published information in the literature on at least five other pathologies [10]: CP [14, 56–58], poliomyelitis [59], traumatic brain injury [60], spinocerebellar degeneration [61] and brain tumour surgery [62]. There is currently no information on usability satisfaction in these five pathologies.

Satisfaction with the use of the exoskeletons was generally positive among users. However, based on the results found, it appears that the exoskeletons need to be optimised to improve the ease of fitting and removal of the device, its weight and size, and its ease of use, among others. Concerning the ease of adjustment, Bryce et al. [63] suggested that individuals should be able to don and doff an exoskeleton independently in five minutes or less. However, other studies indicated that 84% of patients were able to don and doff independently, with a mean of 9:01 and 2:44, respectively [63–65]. The size and weight of the wearable exoskeleton are still heavy and bulky, due to their rigid structures, actuators and batteries. Rodríguez-Fernández et al. [10] report that the average weight of hip-knee exoskeletons is 14.3 kg (7.1 kg/leg), which approximately corresponds to the weight of an average adult human leg (i.e. 10.9 kg) [66]. Finally, the ease of use of the walking exoskeletons is another outstanding aspect to be improved. The next steps in the development of these devices could be directed towards improving usability in non-clinical settings and their access to the community. The transition from hospital facilities to community use requires a well-trained caregiver [67–69]. It is sometimes challenging for people with neurological pathology to identify a caregiver who will dedicate time and effort to support their partner during ambulation with the exoskeleton. Designing systems that do not require, or decrease, reliance on the caregiver could be a future goal for the development of exoskeleton usability in the community. In this review only one exoskeleton is used outside clinical environment, specifically in home and in the community [13]. Most participants do not perceive community use of these devices to be viable as a practical mobility solution. However, they also point to the therapeutic value of robotic exoskeletons in improving many aspects of functionality.

For the transition of exoskeletons out of the community environment, it is essential to review the cost of this equipment. The current price of these devices is high, although it seems to be starting to come down with the emergence of a multitude of start-up companies in the sector and studies demonstrating their effectiveness [68]. The financial cost of the devices was only addressed in a questionnaire, in which 90% of patients strongly agreed that they would like the exoskeleton to be more affordable in order to be able to access it [39]. Related to this, a qualitative review of patient experiences with walking exoskeletons [70] indicated that participants felt that purchasing this technology for personal use was not feasible given the cost; therefore, their access to robotic exoskeletons has typically been through a research project or in a clinical setting [71–73], in line with the research literature.

Regarding therapists’ satisfaction, the lack of evaluation of this parameter is noteworthy [74], as nowadays it is essential to evaluate the therapists’ perception of these devices in order for them to continue to evolve [75, 76]. In addition, it should be noted that currently exoskeletons are mostly indicated for clinical use, so therapists are the potential customers of the companies that manufacture these devices.

As reflected in the results section above, various ways of assessing satisfaction in patients and therapists have been found. Currently, there is no clear way of assessing people’s satisfaction with this type of device. However, among the scales included in this study, there are some that address more diverse dimensions than others, such as the QUEST scale, the Participant Satisfaction Questionnaire scale, the Usability Evaluation Questionnaire of Walking Devices and the REX Acceptability Questionnaire. Considering that the last two were created for a specific study and by a commercial brand focused on a specific exoskeleton model respectively, it is thought that the most appropriate for assessing participant satisfaction at present are the QUEST and the Participant Satisfaction Questionnaire. Furthermore, these two were the most widely used in the included studies. Moreover, it could be interesting in future studies to use both scales, as it could complement the information, since the QUEST scale addresses its items in a more general way and the Participant Satisfaction Questionnaire goes into more specific details of the device use.

It is essential to highlight the importance of ensuring that the satisfaction assessments of both patients and therapists are not influenced by the commercial brands of the exoskeletons, as can be seen in some of the studies included in this review [39, 52], which use self-completed questionnaires designed by the companies that develop the devices. This could introduce biases in the evaluation derived from conflicts of interest of these brands.

In addition, it should be noted that no studies have been found where the satisfaction of the child population and/or their parents with the use of exoskeletons has been assessed. However, there is not much literature available on the use of these types of portable devices in the paediatric population. In the current literature available, to the best of our knowledge, only one overground device in the market has been found to be indicated for the paediatric population [14, 77]. Therefore, robotic exoskeletons should be considered within the developmental stages to assist children with SCI and other clinical populations.

### Limitations and considerations for future studies

We should be cautious in generalizing the results of this study since most of the studies included were Non-Randomized Studies on Intervention (NRSI). According to the Cochrane Handbook, it was decided to include NRSI following Reeves et al. [78] instructions for the inclusion of NRSI and RCT, since the objectives of our work cannot be answered by RCT alone at present. In the current literature, most publications on gait exoskeletons are NRSI, due to the fact that it is a new approach used for gait rehabilitation. Accordingly, all but one study included in this systematic review were published in the last six years.

Thus, further high-quality studies with larger sample sizes are needed to show more evidence in this area. All included studies show positive results of gait exoskeleton therapy satisfaction, but further investigation is needed to show solid conclusions about the efficacy of walking exoskeleton therapy. In addition, it is believed that the questionnaires evaluated, at least for the most part, have not passed the validation process for use in the studies. Therefore, it would be interesting to validate this type of questionnaires in order to increase the reliability of the results.

## Conclusion

The satisfaction of users with gait overground exoskeletons in stroke, SCI and MS seems to show positive results in satisfaction parameters related to the safety, efficacy and comfort of the devices. However, the worst rated aspects and therefore those that should be optimized from the users’ point of view are ease of adjustment, size, weight, and ease of use. However, due to the heterogeneity of procedures to assess satisfaction and the low quality of the studies, the design of studies with high methodological quality and more homogeneous satisfaction outcome measures is recommended.

### Implications for future: a new approach to exoskeletons design

At the moment, for the reasons mentioned above, there are people who still see the field of walking exoskeletons as under development: due to their weight, ease of use, ease of adjustment and removal, their economic cost and their adaptation to different pathologies. But what can be done about this problem?

Nowadays, the physical appearance of exoskeletons is rigid and closed to adaptations. The proposal is to change the trend by creating simpler devices customized to the patient’s functional capacity and therapeutic needs [75]. Therefore, it would be appropriate to take into consideration the proposal of some developers [79] to consider exoskeletons as a set of modules, each one being a single exoskeleton for a joint of the lower extremity (hips, knees and ankles) [80–82]. These systems could be assembled in a specific configuration to respond to a gait deficit in a particular patient. Therefore, therapists or users could choose the configuration of modules needed depending on their pathology and functional capacity. This type of system could provide multiple benefits over the current ones:

1. The weight of the device would be reduced by having only the joints needed in each case, instead of the entire bilateral exoskeleton.
2. By acting only on the joints where the user needs additional force, exoskeleton energy consumption would be optimized. Less current will be consumed by having fewer actuators and therefore the battery will last longer.
3. The active work by the patient is increased by avoiding the support given to healthy joints.
4. By adjusting to the number of joints the user needs, the cost of the device could be proportional to the number of joints needed.
5. For rehabilitation centres or hospitals, a single device could allow them to cover more pathologies, for example: people with complete cervical SCI could use the entire exoskeleton bilaterally; people with stroke could use only the single leg configuration; people with MS could use the exoskeleton using only one knee, only one hip, or only knee and hip without the structure of the entire exoskeleton. Furthermore, if hospitals and rehabilitation centres were to purchase a complete modular exoskeleton, they could have the possibility to treat several patients simultaneously with the same device using different joints, improving the cost-effectiveness of the device.
6. The use and progressive implantation of modules could help reduce the psychological impact of exoskeleton use in degenerative diseases.
7. The time and ease of fitting and removal of the device to the patient could be optimized by only fitting the necessary joints.

Therefore, based on the above and from the clinical and practical point of view, it is thought that the proposed design could have additional benefits and improvements over the current exoskeletons. Nevertheless, the design of a modular exoskeleton requires to pay special attention to some aspects in order to ensure the expected functioning. As the structure of the device may not discharge to the ground in some phases of gait, e.g. in the single knee joint configuration; or in the full cycle, e.g. in the single hip joint configuration; it is necessary for the device to be as light as possible. Furthermore, this weight should be distributed in such a way that it does not affect the symmetry of the step during the walking process, by locating it wherever possible at the waist or trunk. On the other hand, fittings must consider that in any of the configuration’s torque is transmitted effectively and joint alignments are maintained.

## Electronic supplementary material

Below is the link to the electronic supplementary material.

Additional File: Clinical Trial Assessment Identification

## Data Availability

All data generated or analysed during the study are included in this published article and its supplementary information files.

## List of abbreviations

BWSTT: Body-Weight-Supported Treadmill Training
CP: Cerebral Palsy
CSQ-8: Client Satisfaction Questionnaire
DOF: Degrees of Freedom
KAFO: Knee Ankle Foot Orthosis
MAK: Marsi Active Knee
MMCAT: McMasters Critical Appraisal Tool
MS: Multiple sclerosis
N/A: Not applicable
NIH: National Institutes of Health
OCEBM: Oxford Centre for Evidence-Based Medicine
PRISMA: Preferred Reporting Items for Systematic Reviews and Meta-Analyses guidelines
PSQ: Participant Satisfaction Questionnaire
PTSQ: Physical Therapist Satisfaction Questionnaire
QUEST: Quebec User Evaluation of Satisfaction with Assistive Technology
RCT: Randomized Controlled Trial
SCI: Spinal Cord Injury
SUS: System Usability Scale
Th: Thoracic
WOS: Web of Science
